# Perceived causes and risk factors of Buruli ulcer among patients at Agogo Presbyterian hospital in Ashanti Region of Ghana

**DOI:** 10.1186/s13104-018-3172-5

**Published:** 2018-01-23

**Authors:** Reindolf Anokye, Enoch Acheampong, Wisdom Kwadwo Mprah, Edward Sarpong

**Affiliations:** 1 Centre for Disability and Rehabilitation Studies, Department of Community Health, Kwame Nkrumah University of Science and Technology, Kumasi, Ghana; 2 Presbyterian Hospital, Agogo, Ashanti Ghana

**Keywords:** Knowledge, Buruli ulcer patients, Causes and risk factors

## Abstract

**Objective:**

The incidence of Buruli ulcer has been recorded in about 30 countries globally and Africa seems to be the most affected area. The study sought to determine perceived causes and risk factors of Buruli ulcer among patients who visit the Agogo hospital in Asante-Akim North District in the Ashanti region of Ghana. A descriptive study design was adopted using a simple random sampling technique to select 400 patients attending The Presbyterian Hospital at Agogo. Data was collected using a structured questionnaire and analysed using SPSS version 16.0.

**Results:**

Buruli ulcer was perceived as a disease caused by witchcraft (38%), enemies (15%), as well as not pouring libation or praying (16%). Also, increased appetite (30%), oedema or swelling on the skin (29%) and over weight (23%) was perceived as signs and symptoms of Buruli ulcer and a section of the respondents (53%) did not know any risk factor. The age of respondents, gender and level of education were found to determine knowledge of Buruli ulcer (*P* ≤ .05). Public Educations and campaigns should focus on causes and risk factors to ensure that there is adequate knowledge among the general public on Buruli ulcer.

**Electronic supplementary material:**

The online version of this article (10.1186/s13104-018-3172-5) contains supplementary material, which is available to authorized users.

## Introduction

Buruli ulcer dehydrates its victims economically, disfigures one physically and may influence social stigma [1].

Buruli ulcer is caused by *Mycobacterium ulcerans* the bacteria that cause tuberculosis and leprosy [2]. How it is transmitted is still being investigated [3] but the risk factors include proximity to stagnant or slow-flowing bodies of water, poor wound care and not wearing protective clothing [2]. It starts as a painless swelling (nodule) which can ulcerate within 4 weeks causing gross deformities if left untreated [2].

The West African sub region has been mostly affected with Cote d’Ivoire recording 24,000 cases from 1978 to 2006 [2]. About 7000 cases were also reported from 1989 to 2006 in Benin and 51 cases have been confirmed in Nigeria since its discovery [4].

More than 11,000 cases have been reported in Ghana since 1993 with Asante-Akim North, Amansie West District as well as the coastal areas of Ga West District been some of the most endemic areas [5]. In spite of the incidence of the disease in the country, people do not know about it and perceive it as God inflicted disease or caused by drinking from ponds, swimming in rivers, wading in swampy areas, witchcraft and curses [6–8].

Loss of productivity caused by lengthy hospital stay is a concern, as well as the disabling outcomes that frequently occur as a result of Buruli ulcer [9].

Even though Asante-Akim North has the second highest prevalence rate of the disease (prevalence 131.5/100,000) in Ghana [5], various studies conducted on Buruli ulcer knowledge were carried out in Ga West Municipality or Amansie West District [7, 8, 10, 11]. However, a study conducted in Asante-Akim North District on behavioral sanitation impact on *Mycobacterium ulcerans* infection focused mainly on assessing hygienic behaviours among a relatively smaller proportion of respondents [12]. This study differs in terms of its location, study aim and sample size and improves upon previous work on Buruli ulcer in the study area by exploring knowledge on signs, symptoms and prevention of Buruli ulcer that were not explored.

Also, Records at the Agogo hospital indicate that Buruli ulcer cases are mostly reported at the hospital but most cases are presented late which complicates the conditions of patients.

Adequate knowledge about the disease is important in order to increase early detection that may reduce the trauma, pain and cost that comes with late treatment [5].

This study was therefore conducted to determine the perceived causes and risk factors of Buruli ulcer among patients of Presbyterian hospital, Agogo in Asante-Akim North District in the Ashanti region of Ghana.

## Main text

### Methods

#### Study design

This study used descriptive design through quantitative approach which was carried out from November 2015 to May 2016.

#### Study site

The study site was the Presbyterian hospital, in Agogo Asante which is a Municipal hospital in Ashanti-Akim North. It is one of the largest healthcare facilities in Ashanti Region of Ghana and serves as a referral centre for many hospitals. The study site was selected because the Hospital serves as a Training Centre for Buruli ulcer Treatment in Ghana and as Collaborating Centre for the University Of Ghana School Of Public Health. The District is known to be one of the most endemic areas in Ghana when it comes to Buruli ulcer cases [5].

#### Inclusion/exclusion criteria

Inclusion was based on individuals’ status as Buruli ulcer patient and the non-Buruli ulcer patients were selected based on only those who visited the hospital for various reasons. They were selected from both outpatient and inpatient units.

#### Sample size and sampling

A simple random sampling technique was employed to select 400 respondents whose knowledge on the causes and risk factors of Buruli ulcer were assessed using a structured questionnaire. ‘Yes’ and ‘No’ was written on pieces of papers and those who selected the ‘Yes’ were selected for the study. In each particular day and at each particular time that data was collected, patients who were at the hospital were approached and after explaining the purpose of the study to them and obtaining their consent were asked to select one of the papers that had either ‘Yes’ or ‘No’ written in them. Respondents who selected papers that had ‘Yes’ written on it were selected for the study.

The sample size for the study was determined using Yamane [13] formula. For this study, 95% confidence level and the level of precision (P = .05) were used adopting the Equation;

$$n = \frac{N}{{1 + N(e)^{2} }}.$$

The symbol *n* in the equation represents the sample size; *N* represents the population size while *e* represents the level of precision. Agogo Hospital was estimated to have provided services to about 4000 patients with confirmed and suspicious Buruli ulcer cases within the previous 3 years. Using this formula,

$$N=4000$$

$$1 + N (e)^{2} = 1 { } + { 4}000(.05)^{2}$$

$$n = \frac{4000}{{1 + 4000 (.05)^{2} }}$$

$$n=364$$

A 10% non-respondent rate was assumed and therefore 36 were added to 364 to give us a sample size of 400. Data was collected within a period of 3 months and two research assistants were employed.

#### Instrument for data collection and analysis

A structured questionnaire (Additional file 1) was used which was divided into five (5) subsections that includes Demographic information, Knowledge on Buruli ulcer, Knowledge on causes, Knowledge on risk factors and Prevention. The questionnaire was designed based on the objectives that guided the study and was not taken from any previous study.

A pre-test of the questionnaires was conducted at Manso Nkwanta in Amansie West District which is a Buruli ulcer endemic area. The results of the pre-test helped in reshaping the questionnaire for the actual study. SPSS software version 16.0 was used to analyse the data and results were presented using frequency tables.

### Results

#### Demographic characteristics of respondents

Table 1 show that participants were youthful in nature because all of them were between the ages of 15–39 years. Again, 48% were married while 44–8% were single and widowed respectively. More females (51%) than males (49%) took part in the study and 18% of the respondents had no formal education, while 82% had completed at least primary education.

**Table 1:** Demographic Characteristics of respondents. Source: Field survey, 2016

Variables	Characteristics	Frequency	Percentage	
Age (years)				
	15–19	78	20	
	20–24	84	21	
	25–29	92	23	
	30–34	77	19	Mean = 31.3
	35–39	69	17	SD = 12.068
Gender				
	Male	196	49	
	Female	204	51	
Educational background				
	Primary	71	18	
	Middle/JHS	110	27	
	Sec/SHS/A level	80	20	
	Tertiary	69	17	
	No formal education	70	18	
Marital status				
	Married	190	48	
	Single	176	44	
	Widowed	34	8	
Ethnicity				
	Akan	165	41	
	Ewe	75	19	
	Ga/Adangme	58	15	
	Gonja	48	12	
	Mole-Dagbani	54	13	
Occupation				
	Unemployed	85	21	
	Farmer	64	16	
	Civil servant	64	16	
	Trader	109	27	
	Artisan	78	20	

#### Causes and risk factors of Buruli ulcer

The causes and risk factors of Buruli ulcer according to the respondents are as shown in Table 2. From the table, 38% said Buruli ulcer is caused by witchcraft, 15% said enemies, 10% said *Mycobacterium ulcerans*, 7% mentioned ancestors while 30% did not know what causes Buruli ulcer. It can also be observed that more than half of respondents (53%) said they did not know any risk factors of Buruli ulcer, 16% mentioned not pouring libation or praying (offending the gods) 17% believed that having multiple sexual partners is a risk factor, 8% indicated poor personal hygiene 5% said swimming or wading in ponds and while1% mentioned drinking non-potable water as risk factors.

**Table 2:** Showing causes and risk factors of Buruli ulcer. Source: Field work, 2016

Responses	Frequency	Percentages
Causes of Buruli ulcer		
Witchcraft	152	38
Do not know	120	30
Enemies	60	15
*Mycobacterium ulcerans*	40	10
Ancestors	28	7
Total	400	100
Risk factors of Buruli ulcer		
Do not know	212	53
Multiple sexual partners	68	17
Not pouring libation	64	16
Poor hygiene	32	8
Swimming or wadding in ponds	20	5
Drinking non potable water	4	1
Total	400	100

#### Signs, symptoms and prevention of Buruli ulcer

Signs, symptoms and prevention of Buruli ulcer are presented in Table 3 which shows that 30% mentioned an increased appetite as a sign and symptom of Buruli ulcer, 29% mentioned oedema or swelling on the skin, 23% said over weight and 6% mentioned painless plaque of a well-demarcated lesion while 12% said they do not know any sign or symptom.

**Table 3:** Showing signs, symptoms and prevention of Buruli ulcer. Source: Field work 2016

Responses	Frequency	Percentage
Signs and symptoms		
Increase in Appetite	120	30
Oedema or swelling on the skin	116	29
Overweight	92	23
Don’t know	48	12
Painless plaque of a well-demarcated lesion	24	6
Total	400	100
Prevention		
Providing clean water	160	40
Avoiding swimming in the river	100	25
Don’t know	100	25
Education on personal hygiene	40	10
Total	400	100

In terms of prevention, 40% said Buruli ulcer can be prevented through clean water usage, 25% mentioned avoiding swimming in rivers, 10% mentioned education on personal hygiene and 25% had no idea.

### Discussion

The study revealed that tradition and superstition played significant role in explaining what causes Buruli ulcer as most of the respondents believed that Witchcraft, enemies, offending the gods can cause Buruli ulcer. The results implies that majority of the respondents view Buruli ulcer from cultural and traditional perspectives in terms of what causes it and had no idea of the actual causes. There is the potential of increased risk for Buruli ulcer since causes are not known. The effect of this is that people are likely to engage in behaviours that may expose them to possibility of contracting Buruli ulcer without them knowing. The findings on the causes of Buruli ulcer is similar to that of Owusu–Sekyere et al. [11] who also found that in most communities affected by Buruli ulcer in Ghana, witchcraft can be used to inflict others with Buruli ulcer. Similar beliefs have been reported by Adobea and Adamba [8] that people thought Buruli ulcer is caused by their own adversaries, including witches as well as Stienstra et al. [14] study that reported witchcraft as a major cause of the disease perceived by study participants. Also, witchcraft as the cause of the disease was reported by Renzaho et al. [7] with 5.2% of respondents suggesting so. These findings are not surprising since both studies were conducted in the same country (Ghana) where tradition, superstition and culture permeate all aspect of the lives of the people. In terms of risk factors, majority did not know of any risk factor for Buruli ulcer.

The poor knowledge on the risk factors means that the people may indulge in behaviors that may put them at higher risk of contracting the disease. This calls for serious public health education and promotion in order to bring the disease under control. The poor knowledge is similar to the study of Kamga et al. [15] who reported low level of knowledge in his study.

The results indicate that a good number of respondents 30% mentioned an increased appetite as a sign of Buruli ulcer. This finding reveals that respondents had poor knowledge and understanding of the disease. The implication is that early detection and treatment is unlikely. This has the potential of worsening health conditions of individuals which could have been managed if diseases are detected earlier. Despite the cultural and traditional perspectives of viewing Buruli ulcer, a good number of the respondents still believe that good practices at the community level can prevent the spread of Buruli ulcer in the community. The implication is that people may not report to the hospital for appropriate intervention and those who will report are likely to delay and may also be based on wrong assumptions.

Greater proportion of respondents also believed that Buruli ulcer can be prevented when residents are provided with clean water. This suggests that, respondents believed that government has a major role to play in the prevention of the spread of Buruli ulcer in Ghana and in communities such as Agogo. The irony of their suggestion is that, most of them explained the cause of the disease from spiritual perspective yet believed that provision of clean and potable water can help prevent the disease. Since Agogo is an endemic zone for Buruli ulcer, it was expected that at least majority of the respondents would have had a fair idea of how to prevent the disease just like as revealed in a study of Tia et al. [16] where 83% were familiar with the disease.

### Conclusion

Buruli ulcer was perceived as a disease caused by witchcraft (38%), enemies (15%), as well as not pouring libation or praying (16%). Also, increased appetite (30%), oedema or swelling on the skin (29%) and over weight (23%) was perceived as signs and symptoms of Buruli ulcer and a section of the respondents (53%) did not know any risk factor. The attribution to witchcraft and enemies and not knowing the signs and symptoms and risk factors may affect early detection and treatment as most of them will not report signs at a healthcare facility which may worsen their condition. The Age of respondents, Gender and Level of Education were found to determine knowledge of Buruli ulcer (*P* ≤ .05).

## Recommendations

- The Ministry of health, in collaboration with the National Commission on Civic Education should embark on public education and campaigns in endemic areas with special focus on causes and risk factors to improve awareness among the general public on Buruli ulcer.
- The government must also collaborate with the various communication networks in the country to send frequent messages on Buruli ulcer to the general public.
- The Department of Public Health of the Ghana Health Service must develop Buruli ulcer sensitization programmes, hand out brochures and leaflets to inform people and use the various media outlets to improve public knowledge on Buruli ulcer.

## Limitation of the study

The study was limited to one hospital and therefore the findings cannot be generalised. However, the quality of data gathered was not affected in any way by this limitation.

## Additional file

**Additional file 1.** Questionnaire.

## Abbreviations

BU: Buruli ulcer
WHO: World Health Organization
HIV: Human Immunodeficiency Virus
