# Supplementary material for: Perceived causes and risk factors of Buruli ulcer among patients at Agogo Presbyterian hospital in Ashanti Region of Ghana
Source: BMC Res Notes. 2018 Jan 23;11:64. doi: 10.1186/s13104-018-3172-5 (PMC5782383; doi:10.1186/s13104-018-3172-5)
Supplement: Supplementary file 1 — Additional file 1. Questionnaire. [file 13104_2018_3172_MOESM1_ESM.docx]

**Questionnaire**

**Section A: Demographic information**

1. Gender of respondent

Male [ ]

Female [ ]

2. Age

a) 15-20 [ ]

b) 21-25 [ ]

c) 26-30 [ ]

d) 31-40 [ ]

e) More than 40 years [ ]

3. What is your highest level of education?

a. primary [ ]

b. senior secondary [ ]

c. university [ ]

d. no formal education [ ]

4. Marital status:

a) Married [ ]

b) Single [ ]

c) Divorced / separated [ ]

d) Widowed [ ]

5. Ethnicity

a) Akan [ ]

b) Ewe [ ]

c) Ga/Adangme [ ]

d) other (specify)…………………

6. Occupation:

a) Trader [ ]

b) Artisan [ ]

c) Civil servant [ ]

d) Unemployed [ ]

e) Hair dresser [ ]

f)Farmer [ ]

**Section B: Knowledge on Buruli ulcer**

8. What is Buruli ulcer?

a) It is the increase in body temperature [ ]

b) Rashes on the body [ ]

c) infectious disease caused by Mycobacterium ulcerans [ ]

d) infectious disease caused by virus [ ]

e) Don’t know [ ]

**Section C: Knowledge on causes**

9. What do you think can cause Buruli Ulcer?

a) Witchcraft [ ]

b) Ancestors [ ]

c) Mycobacterium ulcerans [ ]

d) Enemies [ ]

e) Other …………

f) Don’t know [ ]

**Section D: Knowledge on risk factors**

10.What are the risk factors of Buruli ulcer ?

a) Drinking non-potable water [ ]

b) Poor personal hygiene [ ]

c) Swimming or wading in ponds [ ]

d) Overeating [ ]

e) Multiple sexual partners [ ]

f) Not pouring libation/praying [ ]

g) Other ……………………..

h) Don’t know [ ]

**Section E: Prevention**

11. How do you think Buruli could be prevented in the community?

a) By providing us with clean water [ ]

b) By avoiding swimming in the river [ ]

c) I do not know [ ]

d) Others (specify) ------------------------------------

12. How can you prevent yourself from being infected with Buruli Ulcer?

a) Praying [ ]

b) Not offending anyone [ ]

c) Vaccination [ ]

d) Not talking to an infected person [ ]
